# Vessel Responses to Thin vs Thick Strut Polymer-Free Biolimus A9-Coated Stents

**DOI:** 10.1016/j.jacasi.2026.01.008

**Published:** 2026-03-06

**Authors:** Masaya Kusuda, Takayuki Ishihara, Isamu Mizote, Daisuke Nakamura, Shumpei Kosugi, Naotaka Okamoto, Tatsuya Shiraki, Naoki Itaya, Takuya Tsujimura, Mitsuyoshi Takahara, Takaharu Nakayoshi, Osamu Iida, Yosuke Hata, Masami Nishino, Takafumi Ueno, Daisaku Nakatani, Shungo Hikoso, Shinsuke Nanto, Toshiaki Mano, Yasushi Sakata, COLLABORATION Investigators

**Affiliations:** a Kansai Rosai Hospital Cardiovascular Center, Amagasaki, Japan; b Department of Cardiovascular Medicine, Osaka University Graduate School of Medicine, Suita, Japan; c Division of Cardiology, Osaka Rosai Hospital, Sakai, Japan; d Division of Cardiovascular Medicine, Kurume University School of Medicine, Kurume, Japan; e Department of Diabetes Care Medicine, Osaka University Graduate School of Medicine, Suita, Japan; f Cardiovascular Division, Osaka International Medical & Science Center, Osaka, Japan; g Seihokai Marine Hospital, Fukuoka, Japan; h Department of Cardiovascular Medicine, Nara Medical University, Nara, Japan; i Department of Cardiovascular Medicine, Nishinomiya Municipal Central Hospital, Nishinomiya, Japan

**Keywords:** cobalt-chromium thin-strut polymer-free biolimus A9-coated stent, coronary angioscopy, optical coherence tomography, stainless-steel polymer-free biolimus A9-coated stent, vessel response

Although the stainless-steel polymer-free biolimus A9-coated stent (SS-BCS; BioFreedom, Biosensors Interventional Technologies, Singapore) has no polymer, it has thicker stent struts (112-120 μm) than other newer-generation stents.^1^ Recently, a next-generation cobalt-chromium thin-strut polymer-free biolimus A9-coated stent (CoCr-BCS; BioFreedom Ultra, Biosensors Interventional Technologies) of 84-88 μm was developed.^2^ However, the vessel responses after CoCr-BCS implantation have not been elucidated. This study aimed to compare the vessel responses between CoCr-BCS and SS-BCS after stent implantation using optical coherence tomography (OCT) and coronary angioscopy (CAS).

The Collaboration-Ultra study is a multicenter (4 centers), prospective, observational study including 55 lesions from 46 patients who underwent percutaneous coronary intervention (PCI) with CoCr-BCS implantation at Kansai Rosai Hospital between June 2022 and February 2024. As a control group, we selected 71 lesions from 51 patients with SS-BCS implantation between August 2018 and February 2020 in the Collaboration-1 study, a multicenter (4 centers) prospective observational study.^3^ OCT and CAS examinations were performed at 12 months after PCI. This study was approved by each hospital’s Ethics Committee and adhered to the Declaration of Helsinki.

The primary outcome was mean neointimal thickness evaluated using OCT. The secondary outcomes were adequate strut coverage rate (neointimal thickness ≥40 μm^3^^,^^4^) on OCT and dominant neointimal coverage (NIC) grade, maximum yellow color grade, and thrombus grade on CAS. The dominant NIC grade was classified as follows:^5^ grade 0, stent struts completely visible; grade 1, struts bulging into the lumen although covered; grade 2, struts were embedded in the neointima but translucently visible; and grade 3, struts completely embedded and invisible. The yellow color grade:^6^ grade 0, white; grade 1, light yellow; grade 2, yellow; and grade 3, intense yellow. The thrombus grade:^7^ grade 0, no thrombus; grade 1, focal (several spotty thrombi); and grade 2, diffuse (thrombus extending between the struts). Linear regression and ordinal logistic model analyses were performed to determine the impact of the CoCr-BCS on OCT and CAS findings at 12 months, respectively.

Overall, 97 patients (126 lesions) were enrolled. At follow-up, target lesion revascularization (TLR) occurred in 3lesions from 3 patients in the CoCr-BCS group and in 2 lesions from 2 patients in the SS-BCS group; OCT and CAS could not be performed for these lesions. Twelve-month follow-up could not be performed for 5 lesions from 3 patients in the CoCr-BCS group and for 5 lesions from 4 patients in the SS-BCS group. In addition, analyzable imaging data were unavailable for 2 lesions in 1 patient in the CoCr-BCS group. Finally, 40 (45 lesions) and 45 (63 lesions) patients in the CoCr-BCS and SS-BCS groups, respectively, underwent a 12-month follow-up. The median follow-up period was 370 days (IQR: 343-392 days).

No significant differences were observed in baseline patient characteristics between the groups. Regarding lesion characteristics, the proportion of type B2/C lesions (American College of Cardiology/American Heart Association classification) was significantly higher in the SS-BCS group (23 of 45 [51%] vs 53 of 63 [84%]; *P* = 0.002). Regarding procedural characteristics, the proportion of predilation was higher in the SS-BCS group (32 of 45 [71%] vs 55 of 63 [87%]; *P* = 0.036), whereas stent length was shorter (median [IQR]: 19 [14-24] mm vs 28 [18-36] mm; *P* = 0.002) and predilation balloon (16 ± 6 atm vs 12 ± 4 atm; *P* = 0.002) and stent implantation pressures (8 ± 2 atm vs 7 ± 3 atm; *P* = 0.006) were higher in the CoCr-BCS group.

In OCT findings before PCI, the proportion of calcified plaque was significantly lower in the CoCr-BCS group (median [IQR]: 0 [0-8.33]% vs 0 [0-50]%; *P* = 0.011). Immediately after PCI, although no significant differences were observed between the 2 groups in terms of lumen or stent area, the incidence of smooth protrusion (45 of 45 [100%] vs 55 of 63 [88%]; *P* = 0.046) and mean embedded distance (median [IQR]: 50.9 [36.3-74.2] μm vs 36 [27-43] μm; *P* = 0.003) were higher in the CoCr-BCS group. OCT findings at 12 months are shown in Figure 1A. The mean neointimal thickness was significantly higher in the CoCr-BCS group. In addition, the adequate strut coverage rate was higher with the CoCr-BCS. CAS demonstrated the dominant NIC grade was higher in the CoCr-BCS group (Figure 1B). No significant differences were observed between the 2 groups in terms of maximum yellow color grade or thrombus grade.

**Figure 1:**
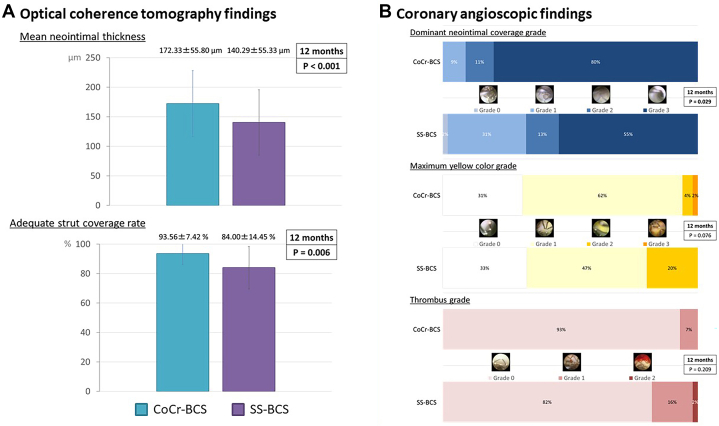
OCT and Coronary Angioscopic Findings at 12 Months (A) Optical coherence tomography (OCT) findings. The mean neointimal thickness was significantly increased in the cobalt-chromium thin-strut polymer-free biolimus A9-coated stent (CoCr-BCS) than in the stainless-steel polymer-free biolimus A9-coated stent (SS-BCS) (172.33 ± 55.80 μm vs 140.29 ± 55.33 μm; *P* = 0.006). The adequate strut coverage rate was higher in the CoCr-BCS than in the SS-BCS (93.56% ± 7.42% vs 84.00% ± 14.45%; *P* < 0.001). Error bars indicate SDs. (B) Coronary angioscopic findings (dominant neointimal coverage [NIC] grade, maximum yellow color grade, and thrombus grade). Dominant NIC grade was higher in the CoCr-BCS than in the SS-BCS (*P* = 0.029). Maximum yellow color grade and thrombus grade were comparable between the 2 groups (*P* = 0.076 and *P* = 0.209, respectively).

The multivariate analysis adjusted for covariates, such as the American College of Cardiology/American Heart Association classification, predilatation, total stent length, stent implantation pressure, proportion of calcified plaque before PCI, smooth protrusion, and mean embedded distance immediately after PCI, showed that the CoCr-BCS had a positive impact on mean neointimal thickness (coefficient: 31.403; 95% CI: 4.174-58.774; *P* = 0.031), adequate strut coverage (coefficient: 10.332; 95% CI: 4.542-16.110; *P* < 0.001), and dominant NIC grade (OR: 3.439; 95% CI: 1.148-10.301; *P* = 0.027).

This study demonstrated that the CoCr-BCS showed increased neointimal thickness, higher adequate coverage rates, and better NIC grades compared with the SS-BCS. Notably, the difference in mean neointimal thickness was approximately 30 μm, which is comparable to the difference in strut thickness between the CoCr-BCS and SS-BCS, suggesting lumen preservation. In addition, the CoCr-BCS showed higher adequate strut coverage rate than the SS-BCS. This finding suggests that re-endothelialization may have progressed more uniformly in the CoCr-BCS group. Low shear stress downstream of thicker stent struts promotes thrombus formation and delays re-endothelialization.^8^ Therefore, thinner struts may promote re-endothelialization more effectively.

Although the influence of stent material cannot be excluded, strut thickness is considered the dominant determinant of neointimal formation. Therefore, the superior arterial healing observed with the CoCr-BCS is most likely explained by its thinner struts. The greater mechanical strength of cobalt-chromium enables the use of thinner struts, which may have contributed to the favorable arterial healing observed in this group.

Finally, this study has limitations, including its observational design and single-center data for the CoCr-BCS group with different study periods. In addition, several lesions were not evaluable due to restenosis, vessel tortuosity, or poor image quality; therefore, the possibility of attrition bias cannot be excluded. However, all PCI procedures were performed under OCT guidance, and baseline characteristics were adjusted, minimizing potential bias. The absence of early (1-month) and long-term (>1-year) follow-up data remains another limitation, indicating the need for further long-term studies.

## Funding Support and Author Disclosures

This study was supported by Biosensors Japan, Tokyo, Japan. The funding source had no role in the study design, patient enrollment, treatment strategy, revascularization procedures or equipment, data collection, analysis, or interpretation. Dr Ishihara has received lecture fees from Kaneka Co., Ltd, Japan and Nipro Corporation, Japan. Dr Mizote has received a scholarship fund from Abbott Medical, Japan. Dr Iida has received remuneration for lectures and advisory board participation from Abbott Medical, Japan and Kaneka Co., Ltd, Japan. Dr Mano has received a research grant from Abbott Medical, Japan. Dr Sakata has received a scholarship fund from Abbott Medical, Japan. All the other authors have reported that they have no relationships relevant to the contents of this paper to disclose.
